# Vascular ultrasound as a follow-up tool in patients with giant cell arteritis: a prospective observational cohort study

**DOI:** 10.3389/fmed.2024.1436707

**Published:** 2024-07-29

**Authors:** Anne C. B. Haaversen, Lene Kristin Brekke, Tanaz A. Kermani, Øyvind Molberg, Andreas P. Diamantopoulos

**Affiliations:** ^1^Department of Rheumatology, Martina Hansens Hospital, Bærum, Norway; ^2^Faculty of Medicine, University of Oslo, Oslo, Norway; ^3^Department of Rheumatology, Hospital for Rheumatic Diseases, Haugesund, Norway; ^4^Department of Rheumatology, University of California, Los Angeles, CA, United States; ^5^Department of Rheumatology, Rikshospitalet, Oslo, Norway; ^6^Division of Internal Medicine, Department of Infectious Diseases, Akershus University Hospital, Lørenskog, Norway

**Keywords:** giant cell arteritis, ultrasound, relapse, follow-up, large vessel vasculitis

## Abstract

**Objectives:**

To evaluate relapses in giant cell arteritis (GCA), investigate the utility of vascular ultrasound to detect relapses, and develop and assess a composite score for GCA disease activity (GCAS) based on clinical symptoms, ultrasound imaging activity, and C-reactive protein (CRP).

**Methods:**

Patients with GCA were prospectively followed with scheduled visits, including assessment for clinical relapse, protocol ultrasound examination, and CRP. At each visit, patients were defined as having ultrasound remission or relapse. GCAS was calculated at every visit.

**Results:**

The study included 132 patients, with a median follow-up time of 25 months [interquartile range (IR) 21]. The clinical relapse rate was 60.6%. There were no differences in relapse rates between GCA subtypes (cranial-GCA, large vessel (LV)-GCA, and mixed-GCA) (*p* = 0.83). Ultrasound yielded a sensitivity of 61.2% and a specificity of 72.3% for diagnosing GCA- relapse in our cohort. In 7.7% of follow-up visits with clinical relapses, neither high CRP nor findings of ultrasound relapse were registered. In comparison, in 10.3% of follow-up visits without symptoms of clinical relapse, there were both a high CRP and findings of ultrasound relapse.

**Conclusion:**

We found moderate sensitivity and specificity for ultrasound as a monitoring tool for relapse in this prospective cohort of GCA patients. The extent or subtype of vasculitis at the diagnosis did not influence the number of relapses. Based on a combination of clinical symptoms, elevated CRP, and ultrasound findings, a composite score for GCA activity is proposed.

## Introduction

Giant cell arteritis (GCA) involves both the cranial and large vessels (LV) (1, 2). The disease presents in three different subtypes: isolated cranial GCA (c-GCA), isolated LV-GCA, and mixed-GCA with involvement of both cranial vessels and LV (1, 3, 4). The relapse rate is known to be as high as 30–60%, depending on the study design and definition of relapse (5–13). Studies have shown that the relapse rates may be lower in patients with c-GCA (11, 14). However, in many studies, the characterization of c-GCA may be inaccurate, given the absence of systematic examination of the supraaortic tree. The European Alliance of Associations for Rheumatology (EULAR) guidelines for the management of large vessel vasculitis recommends regular follow-up and monitoring of GCA disease activity based on symptoms, clinical findings, and systemic inflammation measured by erythrocyte sedimentation rate (ESR) and CRP levels (15). Imaging may confirm a suspected relapse in cases where laboratory markers of disease activity are unreliable or in the long-term monitoring of structural abnormalities (16, 17). There is no consensus on the optimal imaging modality for follow-up of relapses and/or structural abnormalities. Disease activity assessment in patients with GCA can be challenging as some patients may not have clinical symptoms or elevated acute phase reactants despite active disease (18, 19). Additionally, an isolated increase in inflammation markers is non-specific and should not lead to a modification in the immunosuppressive medication. Furthermore, the increasing use of interleukin-6 inhibitors in GCA treatment renders markers of inflammation unreliable (18–20).

There is no gold standard to evaluate disease activity, which leads to an unmet need for a better understanding and validation of GCA relapses. Given the limitations of blood tests and clinical symptoms as markers of GCA relapse, there is an increasing interest in imaging as a monitoring tool for GCA disease activity. Imaging may be helpful in assessing active disease, yet it is important to emphasize that the lack of a gold standard for disease activity evaluation influences any effort to compare any imaging modality with GCA disease activity. Positron emission tomography (PET), magnetic resonance imaging (MRI), and ultrasound may demonstrate findings that could represent active disease (4). However, imaging may show abnormalities in asymptomatic patients with normal inflammatory markers, and it remains unknown whether this can predict the progression of vascular damage (21). While ultrasound is widely used as a diagnostic tool in patients examined for GCA and is now recommended as a first-line imaging modality by both EULAR and the Norwegian Society of Rheumatology (16, 17, 22), it is not clear if it is useful for disease activity monitoring in clinical practice.

Short-term follow-up studies indicate that ultrasound may be valuable, but studies with longer follow-up times are scarce (12, 23, 24).

In Takayasu arteritis (TAK), which is a rare LV vasculitis primarily afflicting young women, disease activity has since 1994 been evaluated by a composite score including four domains: clinical symptoms, ischemic symptoms, ESR, and LV imaging by angiography (25). While it has been suggested that this composite Takayasu activity score, called the Kerr’s or National Institute of Health (NIH) criteria, could be useful in GCA, it has not been validated in any GCA population. In Norway, a modified version of Kerr’s criteria is used to evaluate disease activity in patients with GCA (22). There remains an unmet need for tools to assess disease activity in GCA better, and given the different subsets, it is likely that a composite score utilizing multiple different domains will be more sensitive than relying on any one measure alone.

The aims of this prospective cohort study were: (i) To assess the number of relapses in GCA patients, including by disease subtype. (ii) To evaluate the utility of ultrasound as a monitoring tool in patients with GCA. (iii) To develop a composite score for measuring disease activity in patients with GCA.

## Methods

### The prospective GCA cohort

Patients with new-onset GCA referred to the Department of Rheumatology, Martina Hansens Hospital in Bærum, Norway, from September 2017 and prospectively followed until September 2022 were included. Patients with confirmed GCA were included in this study. The diagnosis was based on clinical manifestations (headache, jaw claudication, visual disturbance/vision loss, scalp tenderness, bilateral aching of the shoulder girdle and stiffness, limb claudication, and/or constitutional symptoms like fever, fatigue, or weight loss) and imaging findings. All the patients were classified using the ACR 1990 criteria modified by Dejaco et al. (21). The diagnosis was again reassessed and confirmed 12 months later. All the patients were examined at diagnosis using the extended A2-US method. The Extended A2-US method consists of a continuous ultrasound visualization of the large supraaortic vessels (common carotid, vertebral, segment 1–4, the whole subclavian in the right side and the distal part of subclavian in the left side, axillary proximally, and axillary distally including the proximal brachial artery).

Ultrasound examination was performed using high-end equipment, and the sonographers (ACBH, APD) were experienced (APD > 5,000 vascular scannings) and trained (ACBH) according to a standardized program (23). The examination utilized a General Electric S8 ultrasound machine with a 9–12 MHz linear probe for the large vessels, an 8–18 MHz hockey stick probe for the cranial arteries, or a Canon Aplio 800 ultrasound machine with a 3–11 MHz linear probe for the large vessels, and a 8–22 MHz hockey stick probe for the cranial arteries. We used B-mode for all vessels and in addition color Doppler (PRF range 2–3.5 kHz) for the cranial arteries. Colour gain was adjusted according to noise level with a minimum of blooming. Focus was at the level of interest. Frequency for Doppler was the highest possible. The characteristics of this GCA cohort have been described in a previous paper (26). The follow-up included a monthly visit until remission was achieved and then at months 3, 6, 12, and yearly thereafter. Several visits were postponed because of the Covid-restrictions in the period 2020–2022. Patients suspected of having a relapse were evaluated in an unscheduled visit. Data collected at each visit included assessment of clinical relapse (see below), ultrasound examination, serum CRP measurements, Prednisolone dose, and use of other immunosuppressive agents.

### Definitions of GCA relapse

At each visit, data on patients’ symptoms were collected. The consultant rheumatologist clinically classified the patient as having a relapse or being in remission according to the EULAR definitions of key symptoms and clinical findings suggestive of active disease (15). The definitions are shown in Table 1. Per the EULAR recommendations, we considered a clinical relapse (Table 1), the gold standard definition of GCA relapse, and all other parameters were compared to the clinical relapse.

**Table 1:** Definitions of clinical remission and relapse, and definitions of remission and relapse by ultrasound.

Clinical remission	No clinical signs and symptoms attributable to active vasculitis (morning stiffness of the shoulder or neck, sudden visual loss, jaw claudication, headache, scalp tenderness, constitutional symptoms, limb claudication).
Clinical relapse	Clinical signs and symptoms attributed to vasculitis (morning stiffness of the shoulder or neck, sudden visual loss, jaw claudication, temporal headache, scalp tenderness, constitutional symptoms, limb claudication) after a period of clinical remission.
Ultrasound relapse	Involvement of new arterial segments (cranial, temporal, fascial) and/or LV (carotid, vertebral, subclavian, axillary proximal, axillary distal) or augmentation of the IMT ≥0.2 mm in the already involved arteries (LV) compared to the ultrasound findings at the previous visit.
Ultrasound remission	No involvement of new arterial segments (cranial, temporal, fascial) and/or LV (carotid, vertebral, subclavian, axillary proximal, axillary distal) or augmentation of the IMT ≥0.2 mm in the already involved arteries (LV) compared to the ultrasound findings at the previous visit.

### Ultrasound examinations

The patients were examined by extended ultrasound using the extended anteromedial method (A2-US) by the consultant rheumatologist, who was also experienced in vascular ultrasound (26). A halo and compression sign were considered positive findings for the temporal and facial arteries (27). The presence or absence of a halo sign in the cranial and vertebral arteries and the maximum intima-media-thickness (IMT) (thickest area visualized, upper or lower vessel wall) in the supraaortic arteries, assessed by ultrasound, was recorded for all arterial segments at every visit. The patients were classified as having ultrasound findings consistent with ultrasound remission or ultrasound relapse (as defined in Table 1) for every visit during the follow-up period. Settings of ultrasound equipment are presented in previously published papers (4, 26). To compare the ultrasound extent of vasculitis at diagnosis and the number of clinical relapses, we evaluated a combination of halo counts based on the halo score (but not similar) (28): 1*. Simple halo count* (1 point for each temporal, 1 point for each facial and 1 point for every large vessel involved), 2. *Extended halo count* (1 point for every cranial branch involved temporal common, temporal, frontal, temporal parietal, facial) and 1 point for every large vessel involved (carotid, vertebral, subclavian, axillary proximal, axillary distal), and 3. *Modified extended halo count* (1 point for every cranial branch involved temporal common, temporal, frontal, temporal parietal, facial) and 2 points for every large vessel involved (carotid, vertebral, subclavian, axillary proximal, axillary distal).

### GCA disease activity score

We assessed GCA disease activity at diagnosis and every visit by a preliminary composite GCA disease activity score (GCAS) based on the LV vasculitis NIH criteria (25) and modified as follows: 1. Clinical symptoms (features of vascular ischemia or inflammation and/or systemic features) not attributable to conditions other than GCA; 2. Elevated CRP (> 5 mg/L, which is the usual upper reference limit in Norway) not attributable to conditions other than GCA; 3. Positive imaging (ultrasound or other imaging modalities) (involvement of new arterial segments or augmentation of the IMT in the already involved arteries). The cut-off for active disease was ≥2 of 3 criteria.

### Statistical analysis

The chi-square test was used to measure categorical variables. Phi-test, ANOVA, and ANCOVA regression analysis were used for continuous variables, and the phi-test was used to analyze correlations. Statistical analyses were performed using SPSS, version 21 SPSS Inc., Chicago, IL. *p* < 0.05 were considered to be significant.

### Ethical considerations

According to the Declaration of Helsinki, written informed consent was obtained from all participating patients. The Ethics Committee of South-Eastern Norway (Regional Etisk Komite Sør-Øst) approved the study.

## Results

The study included 133 patients with GCA, one patient (subtype LV-GCA) dropped out before the first follow-up visit. For the rest 132 patients, 65.9% female, the mean (SD) age was 72.8 (8.8) years at diagnosis. 31.1% were classified as c-GCA, 15.2% as LV-GCA, and 53.8% as mixed-GCA. The total follow-up time was 3,406 patient months. The median follow-up time was 25 months (IR 21 months), and 80 (60.6%) patients suffered at least one clinical relapse during the study period (Table 2). There was no significant difference in duration of follow-up between GCA subtypes; mean follow-up time for c-GCA 24.7 months (CI 95% 21.1–28.3), for LV-GCA 30.1 months (CI 95% 25.3–34.8), and for mixed-GCA 26.3 months (CI 95% 23.2–29.5) (*p* = 0.27).

**Table 2:** Characteristics of the GCA cohort.

	Patients with clinical relapse (*n* = 80)	Patients without clinical relapse (*n* = 52)	Total	*p*-value
Gender				0.9
Men, n(%)	27(20.5)	18(13.6)	45(34.1)	
Female, n(%)	53(40.2)	34(25.8)	87(65.9)	
Age at diagnosis, years, mean (SD)	72(9.3)	73(7.8)	72.8(8.8)	0.3
Follow-up time, months, median (IR)	**28.5(21)**	**23(17)**	**25 (21)**	**0.015**
Number of visits, median (IR)	**7(4)**	**4(2)**	**6(4)**	**<0.05**
Subtype GCA, n(%)				0.8
c-GCA	26	15	41(31.1)	
LV-GCA	11	9	20(15.2)	
Mixed-GCA	43	28	71(53.8)	
Use of DMARD, n(%)	**52(65.0)**	**11(21.2)**	**63(47.7)**	**<0.005**
Ultrasound relapse ever, yes/no	**75/6**	**30/21**	**132**	**<0.05**
CRP > 5 mg/L during follow-up, yes/no	**69/12**	**33/18**	**132**	**0.006**
Prednisolone dose initially mg, median (IR)	**40 (20)**	**40 (0)**	**40(19)**	**0.02**

SD, standard deviation; IR, interquartile range; GCA, giant cell arteritis; c-GCA, cranial GCA; LV-GCA, large vessel GCA; DMARD, disease modifying drug; CRP, C-reactive protein.

Bold values are statistically significant.

**Figure 1:**
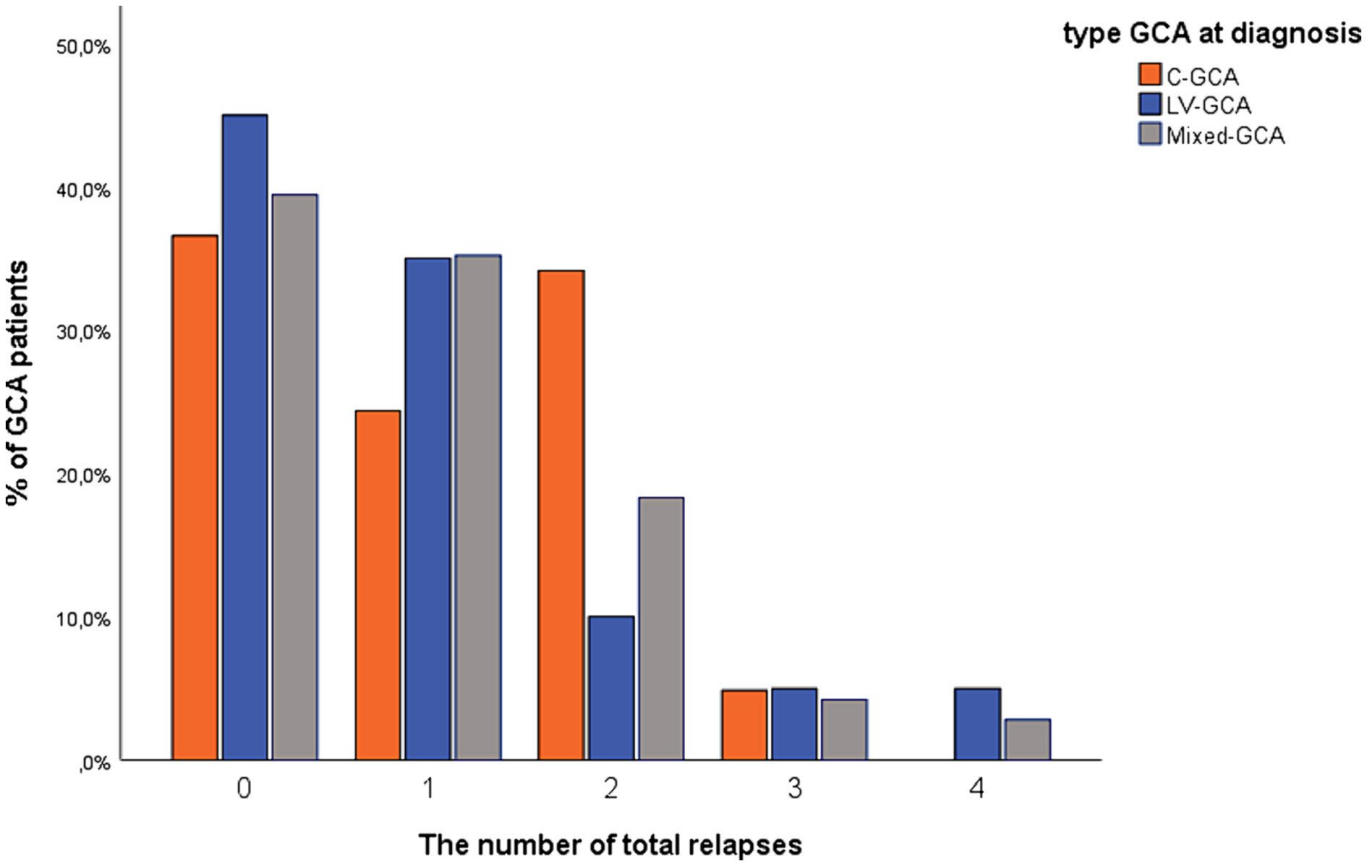
Percentage of GCA patients in the three different subtypes with registered clinical relapse in the study period. c-GCA, cranial-GCA; LV-GCA, large vessel-GCA.

There were no statistical differences with regards to clinical relapse between the different GCA subtypes (c-GCA 26/41 patients (63.4%), LV-GCA 11/20 patients (55.0%) or mixed- GCA 43/71 patients (60.6%) (*p* = 0.82)), or among cranial isolated (63.4%) versus LV involvement 54/91 patients (59.3%) (*p* = 0.83) or between cranial vessel involvement 69/112 patients (61.6%) or LV isolated (55.0%) (*p* = 0.58) (Figure 1).

### Extent of vasculitis by ultrasound at diagnosis and clinical relapse rates

To evaluate whether the extent of vasculitis by ultrasound at diagnosis had any impact on clinical relapse rates during follow-up, we compared the number of vessels having ultrasound findings consistent with vasculitis (defined by halo count-scores at diagnosis) with rates of clinical relapse corrected for time of follow-up. No difference was observed between the extent of vasculitis at diagnosis and the occurrence of a clinical relapse (Table 3), indicating that the extent of vessel involvement is not a predictor for relapse.

**Table 3:** Influence of different halo count-scores of disease extension at diagnosis on clinical relapse during follow-up.

	Status during follow-up		
Ultrasound findings at diagnosis	Clinical relapse (*n* = 139)	Clinical remission (*n* = 581)	*p*-value
Simple halo count, mean (SD)	4.5 (2.6)	4.9 (2.6)	0.35
Extended halo count, mean (SD)	6.2 (3.3)	6.8 (3.2)	0.31
Modified extended halo count, mean (SD)	8.7 (5.4)	9.7 (5.2)	0.22

SD, standard deviation.

### Use of disease-modifying antirheumatic drugs

Of the 132 patients, 63 (47.7%) used DMARDs during the follow-up period, 34.1% of all patients with c-GCA (*p* = 0.06), 65% of all patients with LV-GCA (*p* = 0.03), and 50.7% of all patients with mixed-GCA (*p* = 0.09). The most used immunosuppressive agents were Methotrexate (48 patients) and Leflunomide (15 patients). Seven patients switched to a 2nd DMARD (4 MTX, 2 Leflunomide, 1 Tocilizumab), while 2 patients switched to a 3rd DMARD (1 Tocilizumab, 1 Azathioprine).

### Ultrasound findings at follow-up visits

During the study period, 750 follow-up visits were registered. In 30 follow-up visits (4.0%), ultrasound was not performed, resulting in 720 follow-ups included in the analyses. In 474 (65.8%) visits, ultrasound findings were consistent with our definition of ultrasound remission. In 246 visits, ultrasound findings were consistent with relapse. Ultrasound relapse was isolated to the cranial arteries in 73 visits (29.7%), isolated to LV in 134 visits (54.5%), and involved both cranial and LV in 39 visits (15.9%).

### Comparison of clinical relapse and ultrasound relapse

Of the 139 follow-up visits with clinical relapse, 85 visits had ultrasound relapse as well. In 161 of 581 visits in which patients were in clinical remission, there was evidence of ultrasound relapse, yielding a sensitivity of 61.2% and a specificity of 72.3% for ultrasound relapse in GCA patients. A weak to moderate positive correlation of 0.28 was calculated (*p* < 0.01) among the clinical and ultrasound relapse with a cut-off for LV involvement of 0.2 mm. When the cut-off was raised to 0.3 mm, the correlation increased to 0.3 (*p* < 0.01) and the specificity was raised to 79.0%, while the sensitivity fell to 54.5%. Raising further the cut-off to 0.4 mm reduced sensitivity to 44.0% and raised specificity to 83.0% with an unchanged correlation of 0.3 (*p* < 0.001).

### Clinical relapse and CRP

A total of 615 follow-up visits included CRP values, with CRP >5 mg/L in 281 visits. Median CRP for visits with clinical relapse was 13 (IR 24) mg/L. The correlation of CRP with clinical relapse was 0.28 (*p* < 0.01). Ultrasound activity had a weak correlation of 0.15 with CRP (*p* < 0.01).

### Clinical activity and GCA disease activity score

In 105 visits, CRP measurement was missing; hence, GCAS could be calculated for 615 visits. Ultrasound relapse and CRP > 5 mg/L, together with combinations of them, are compared to clinical relapse in Table 4. One hundred and seventy visits were scored with a positive GCAS (≥2), while 445 visits were GCAS negative (<2).

**Table 4:** Ultrasound relapse and CRP > 5 mg/L compared to clinical relapse.

GCAS component	Follow-up, clinical remission visits (*n* = 485)	Follow-up, clinical relapse visits (*n* = 130)	Sensitivity	Specificity
CRP > 5 mg/L, *n* = 281(%)	189(67.3)	92(32.7)	70.8	61.0
Ultrasound relapse, *n* = 246(%)	161(65.4)	85(34.6)	61.2	72.3
CRP > 5 mg/L AND ultrasound relapse, *n* = 123(%)	66(53.7)	57(46.3)	43.8	86.4
CRP > 5 mg/L OR ultrasound relapse, *n* = 376(%)	262(69.7)	114(30.3)	82.0	45.9

GCAS, giant cell arteritis activity score; CRP, C-reactive protein.

In 10 (7.7%) follow-up visits with clinical relapses, there were neither CRP >5 mg/L nor ultrasound relapses (GCAS<2), and in 50 (10.3%) follow-up visits with clinical remission, there were both CRP>5mg/L and ultrasound relapse (GCAS ≥ 2) (Table 5).

**Table 5:** GCAS compared to clinical relapse.

		GCAS ≥ 2		Total
		No	Yes	
Clinical relapse	No	435	50	485
	Yes	10	120	130
Total		445	170	615

GCAS, giant cell arteritis activity score.

## Discussion

The moderate sensitivity and specificity of ultrasound in diagnosing clinical relapse in GCA patients are the main findings of our study. To our knowledge, this is the first study addressing the use of ultrasound as a follow-up tool in a prospective cohort of GCA patients during a long period, which included both the cranial and the majority of the supraaortic large vessels. Prior research on vascular ultrasound’s role in monitoring GCA has been limited, and mainly on the correlation between disease activity and the presence of a temporal artery halo for a short period or by monitoring small groups of patients and few supraaortic vessels. One prospective study highlighted the ultrasound halo sign’s potential in monitoring GCA. However, it demonstrated sensitivity mainly in temporal arteries without significant findings in the axillary halo regarding disease activity or clinical remission (12). This study included only 6 months of follow-up, and few of the patients in this cohort had axillary involvement (11/49 patients), which poorly reflects the distribution of patients of different subtypes of GCA (12). In a Danish study, different vascular ultrasound scores were evaluated for sensitivity to change (24). They reported that scores containing TA were sensitive to change, while LV responded poorly (24). However, they evaluated only the distal part of the axillary artery and the carotid artery [rarely involved in GCA (26)]. In addition, the follow-up time for most patients was only 24 weeks. Other smaller studies have demonstrated the persistence of axillary halos compared to TA halos, irrespective of clinical remission (12, 23, 29–31).

CRP levels have previously been identified as a marker of low reliability for GCA disease activity due to its lack of specificity and sensitivity, and this was confirmed in our study as well (8, 32, 33). The relapse rate in our study was in the upper range (60.6%) compared to reported in other studies (5–12). This could be explained by the prospective design, the extended follow-up time, and the high rate of DMARD use (47.7%). Our study is the first in which the patients were classified into three groups (cranial-GCA, LV-GCA, and mixed- GCA) by performing ultrasound at diagnosis and following them by ultrasound of both cranial and LV for an extended period. Conflicting data are published on relapse rates in patients with LV versus cranial involvement, with some studies showing that patients with LV involvement did not relapse more often than patients with c-GCA (34, 35), while other studies found that patients with LV involvement relapse more often and earlier than the patients with cranial- GCA (11, 14, 36–39). One reason could be that the majority of studies lack an extended baseline visualization of supraaortic large vessels, thus missing a significant proportion of patients with LV involvement (14, 35, 38, 39).

In our study, we did not observe any influence in the number of relapses of the extent of vasculitis at diagnosis, by using halo count and modifications and corrected for follow-up time in the different groups. A recently published study prospectively performing PET-CT or MRI at the time of treatment discontinuation found no significant difference in the number of vasculitic vessel segments on imaging on relapse rate (40).

The use of DMARDs was observed in 47.7% of the patients in our cohort. Methotrexate was the most used DMARD. This could be explained by The Norwegian Tender System which requires the use of Methotrexate as the first-line Prednisolone-sparing agent in GCA patients (22). In our cohort, only two patients were treated with Tocilizumab—one as a second DMARD switch and the other as a third DMARD switch, with the second patient initiating Tocilizumab treatment very late during the follow-up period. Consequently, the follow-up duration was limited after the initiation of Tocilizumab. This results in a small sample size and insufficient follow-up time, compromising the accuracy and reliability of any comparisons with the Methotrexate group concerning relapse rates.

A greater proportion of patients with LV disease received additional immunosuppressive therapy compared to those with c-GCA, which aligns with previously published data (14).

Interestingly, we did not observe any significant differences in the number of clinical relapses between patients with isolated cranial arteritis and those LV involvement. This finding is somewhat unexpected given the severity often associated with LV involvement. One possible explanation for this observation is the early introduction of DMARDs in patients exhibiting LV involvement. Previous studies have demonstrated that patients with LV involvement typically require higher doses of corticosteroids and have higher relapse rates compared to those with cranial arteritis alone (11, 14). This evidence likely influenced our clinical practice, leading to the proactive and early administration of DMARDs in the LV group. Specifically, Methotrexate was more frequently employed in patients with LV involvement as an adjunct to corticosteroid therapy. Despite these proactive measures, it is important to note that the absence of a significant difference in relapse rates between the two groups may also reflect the heterogeneity of the disease and the complexity of managing GCA. The similar initial corticosteroid doses observed in both groups (44 mg in the LV group vs. 44.13 mg in the cranial group, *p* = 0.9) further support that the baseline treatment approach was comparable, thereby underscoring the potential impact of early DMARD intervention in achieving comparable relapse rates. Thus, our findings suggest that early and aggressive management with DMARDs in patients with LV involvement may mitigate the expected higher relapse rates typically associated with this subgroup. This hypothesis warrants further investigation in larger, prospective studies to confirm the benefits of early DMARD introduction and to refine treatment protocols for GCA patients with varying patterns of vascular involvement.

Several quantitative scores have been developed for using ultrasound in GCA patients (23, 41, 42). Our study employs a method focusing on the development of vasculitic changes in new vascular beds or increased IMT in previously involved vessels. This approach aims for efficiency in clinical practice, contrasting with the time-consuming quantitative scores designed for research purposes. The provisional OMERACT ultrasonography score (OGUS) was shown to have a high sensitivity to change between baseline and follow-up for 24 weeks. Still, the only LV assessed was the axillary artery (6 of 8 arterial segments included were cranial) (42). Most importantly, the calculation of this score is meant for use in clinical trials and not for daily clinical practice. The same applies to the score used in the GUSTO trial, which was also time-consuming/complicated to calculate (23).

There are no well-defined and clinically used outcome measures regarding disease activity in patients with GCA. In the present study, the combination of ultrasound and CRP to judge disease activity yielded high sensitivity and low specificity (ultrasound relapse OR CRP >5 mg/L positive) and low sensitivity and high specificity (ultrasound relapse AND CRP >5 mg/L positive). Interestingly, in TAK, the combination of inflammatory markers, imaging, claudication, and clinical symptoms has been used as an outcome measure for the disease activity (active disease >2 positive components) (43–46). Based on the findings in the present study and the experience with a similar disease (TAK), we propose the combination of clinical symptoms, CRP, and ultrasound findings of activity into a composite score (GCAS) in which two positives of three components indicate active disease. Coath and Mukhtyar used modified NIH criteria (constitutional symptoms, claudication symptoms, CRP >10 mg/L, ultrasonographic changes of GCA) as an aid to diagnose relapse in GCA (31). The Norwegian Society of Rheumatology recommends using NIH criteria to register disease activity (22).

Major strengths of this study are the prospective design, the long-term follow-up with a systematic ultrasonographic protocol, the standardized collection of the follow-up data, the high total number of follow-up visits, and the real-life setting of an outpatient clinic. The ultrasound examination followed a predefined protocol for visualizing blood vessels, and all scans were performed by experienced ultrasonographers using high-end ultrasound equipment. The use of the extended anteromedial ultrasound examination with visualization of all supraaortic vessels (both upper and lower wall) ensured that all vasculitic changes in the vessel wall were visualized and measured (26). Another strength is that our cohort also includes patients diagnosed with LV disease and that the patients were classified into three different subgroups for the very first visit, in contrast to many other studies, which mainly included patients with cranial involvement based mainly on biopsy.

A main limitation is that temporal and facial artery halo were assessed only as present/absent instead of assessment of ultrasound-specific halo features, like the number of segments with halo sign involved and the halo IMT. Another significant limitation is the absence of a universally accepted gold standard for defining relapse in GCA. Our study utilized the EULAR relapse definition, which is based exclusively on clinical criteria. This approach lacks a comprehensive perspective that includes imaging modalities such as PET, MRI, and ultrasound, which are increasingly used to identify relapses. However, as our study suggests, interpreting results from these imaging techniques can be challenging, and many patients may exhibit changes without showing clear signs of active disease. Ultrasonographic changes may persist in some cases in patients thought to be in clinical remission (47, 48). The ultrasonographers were not blinded to the clinical data and CRP level and thus could have been influenced by this information.

In conclusion, ultrasound demonstrates moderate sensitivity and specificity as a monitoring tool in the follow-up of GCA patients. The extent of vasculitis at the diagnosis did not influence the number of relapses in GCA patients, and no difference was seen in relapse rates regarding different GCA subtypes. A composite GCA activity score, combining clinical observations, CRP levels, and ultrasound findings, may be useful to guide the management of this complex condition.

## Data availability statement

The raw data supporting the conclusions of this article will be made available by the authors, without undue reservation.

## Ethics statement

The studies involving humans were approved by the Ethics Committee of Southeastern Norway (REK SørØst). The studies were conducted in accordance with the local legislation and institutional requirements. The participants provided their written informed consent to participate in this study. Written informed consent was obtained from the individual(s) for the publication of any potentially identifiable images or data included in this article.

## Author contributions

AH: Writing – original draft, Writing – review & editing. LB: Writing – original draft, Writing – review & editing. TK: Writing – original draft, Writing – review & editing. ØM: Writing – original draft, Writing – review & editing. AD: Writing – original draft, Writing – review & editing.
