# Correction: Vascular ultrasound as a follow-up tool in patients with giant cell arteritis: a prospective observational cohort study

**DOI:** 10.3389/fmed.2025.1635905

**Published:** 2025-06-12

**Authors:** Anne C. B. Haaversen, Lene Kristin Brekke, Tanaz A. Kermani, Øyvind Molberg, Andreas P. Diamantopoulos

**Affiliations:** ^1^Department of Rheumatology, Martina Hansens Hospital, Bærum, Norway; ^2^Faculty of Medicine, University of Oslo, Oslo, Norway; ^3^Department of Rheumatology, Hospital for Rheumatic Diseases, Haugesund, Norway; ^4^Department of Rheumatology, University of California, Los Angeles, CA, United States; ^5^Department of Rheumatology, Rikshospitalet, Oslo, Norway; ^6^Division of Internal Medicine, Department of Infectious Diseases, Akershus University Hospital, Lørenskog, Norway

**Keywords:** giant cell arteritis, ultrasound, relapse, follow-up, large vessel vasculitis

In the published article, the **Reference** for ACR 1990 criteria modified by Dejaco et al. was incorrectly written as “Maz M, Chung SA, Abril A, Langford CA, Gorelik M, Guyatt G, et al. American College of Rheumatology/Vasculitis Foundation guideline for the Management of Giant Cell Arteritis and Takayasu Arteritis. *Arthritis Rheumatol*. (2021) 73:1349–65. doi: 10.1002/art.41774.”

It should be “Dejaco C, Duftner C, Buttgereit F, Matteson EL, Dasgupta B. The spectrum of giant cell arteritis and polymyalgia rheumatica: revisiting the concept of the disease. *Rheumatology* (2017) 56:506–515. doi: 10.1093/rheumatology/kew273.”

In the published article, there was an error. There was an error in how the modified ACR 1990 criteria were named and referenced.

A correction has been made to **Methods**, *The prospective GCA cohort*, paragraph 1. This sentence previously stated:

“All the patients were classified using the ACR 1990 criteria modified by Maz et al.”

The corrected sentence appears below:

“All the patients were classified using the ACR 1990 criteria modified by Dejaco et al.”

The authors apologize for these errors and state that this does not change the scientific conclusions of the article in any way. The original article has been updated.

